# Phytochemical profile and *in vitro* antioxidant activity of *Emelia M* (EMB), *Mshikazi* and *Delosma H* herbal medicines as demonstrated in THP-1 and Jurkat leukaemia cell lines

**DOI:** 10.4314/ahs.v21i4.51

**Published:** 2021-12

**Authors:** Joy Nkechinyere Adeniyi, Manimbulu Nlooto, Mlungisi Ngcobo, Roshila Moodley, Exnevia Gomo

**Affiliations:** 1 Traditional Medicine Laboratory, School of Nursing and Public Health, College of Health Sciences, University of KwaZulu-Natal; 2 Department of Pharmacy, School of Health Care Sciences University of Limpopo; 3 Department of Chemistry, School of Chemistry & Physics University of KwaZulu-Natal

**Keywords:** Antioxidants, herbal medicines, *Emelia M*, *Mshikazi*, *Delosma H*

## Abstract

**Background:**

Three decoctions, namely Emelia M (EMB), Mshikazi and Delosma H are used by traditional health practitioners in KwaZulu-Natal, South Africa to treat and manage leukaemia and related conditions

**Objectives:**

This study evaluated the in vitro antioxidant activity and phytochemical profile of the aqueous extracts of Emelia M (EMB), Mshikazi and Delosma H decoctions.

**Methods:**

Antioxidant activity of the extracts was evaluated using1-diphenyl-2-picrylhydrazyl (DPPH), glutathione (GSH), phosphomolybdate and thiobarbituric acid reactive substance (TBARS) assays. Phytochemical screening was used to determine the presence of compounds.

**Results:**

The DPPH radical scavenging activity was similar to ascorbic acid for EMB and Delosma H, but not for Mshikazi. At 24 h, EMB increased GSH in both THP-1 and Jurkat cells similar to Delosma H while Mshikazi demonstrated the lowest activity. At 48 h, EMB and Delosma H revealed increased GSH in THP-1 cells with no significant decrease in GSH levels in Jurkat cells. However, EMB showed the lowest lipid peroxidation activity compared to Delosma H and Mshikazi after 24 h treatment of both cells. Phenols, flavonoids, terpenoids, saponins were present in all extracts.

**Conclusion:**

Extracts of the three decoctions possess both antioxidant and prooxidant properties through high scavenging activity and increased in lipid peroxidation.

## Introduction

Oxidative stress (OS) is known as a physiological disturbance in which a high level of free radicals (reactive oxygen species (ROS)) are generated as a result of an imbalance in antioxidant metabolism^1^,^2^. Oxidative stress plays a role in human pathogenesis diseases^1^ and affects all the stages of the oncogenic process starting from the initiation stage all through to the progression stage.^3^ Cells use oxygen to generate energy and free radicals are formed as a result of adenosine triphosphate (ATP) production by the mitochondria. Free radicals are by-products of cellular metabolism^4^,^5^. When ROS are low or moderate they exert an advantageous effect on cellular response and immune function, however, at high levels, they generate oxidative stress which can cause membrane damage^6^, DNA molecule damage, alter signalling pathways and cause progression of various cancers^1^,^7^.

Antioxidants play a key role in the maintenance of good health^8^. The human body has diverse ways to counter oxidative stress, either by producing antioxidants through endogenous (naturally in the body) or exogenous (food and/or synthetic form through supplements) ways. Both endogenous and exogenous antioxidants scavenge free radicals by inhibiting and repairing the damage induced by ROS and hence, boost the immune function and lower the risk of cancer^7^. On the other hand, prooxidants are endobiotic or xenobiotic chemicals that cause oxidative stress either by generating ROS or by preventing antioxidant systems. These include the reactive, free radical containing molecules in cells or tissues^9^.

Plants are sources of a wide range of therapeutic molecules and hence hold a great value for new drugs^10^. Antioxidants such as ascorbic acid, β-carotene, glutathione, α-tocopherol hormones and internal source of enzymes scavenge free radicals thereby protecting the body from oxidative stress. Today various synthetic antioxidants such as hydroxyanisole and butylated hydroxytoluene are commonly included in foods produced in food industries. However, some of the synthetic antioxidants are toxic or have adverse side effects^5^. To substitute the synthetic antioxidants, interest has increased in finding new natural antioxidants for food and medical purposes^5^.

Medicinal plants are a potential source of natural products which have different biological activity^5^ including antioxidant activity^10^. Herbal medicines have shown important benefits by combating oxidative-mediated diseases through several constituents that synergistically work together through various molecular targets for better efficacy^11^. Several studies and reviews have shown that some herbal medicines have been used to manage and treat oxidative-mediated diseases such as cancer, diabetes and cardiovascular disease^12^–^15^. For instance, Trigonella foenum-graecum, Atriplex halimus, Olea europaea, Urtica dioica, Allium sativum, Allium cepa, Nigella sativa, and Cinnamomum cassia were reported to possess anti-diabetic properties and through meiating glucose transporter-4 (GLUT4) to the plasma membrane^16^. Also, Centella asiatica was shown to effectively modulate antioxidant activity, inflammatory cytokines and cell death. In THP-1 cells and peripheral blood molecular cells (PBMCs), it also decreased pro-inflammatory cytokine levels and increased anti-inflammatory cytokine levels which may alleviate cancer cachexia^17^.

Emelia M, Mshikazi, Delosma H are traditional herbal medicines in an aqueous form prepared and used by traditional health practitioners in KwaZulu-Natal, South Africa for the treatment of leukaemia and other related diseases. This study evaluated the in vitro antioxidant activity of aqueous extracts of the herbal medicines and determined their phytochemical profile.

## Materials and Methods

### Reagents and equipment

RPMI-1640 medium supplemented 1% L-glutamine (Cat No. BE-12-702F) was purchased from Whitehead Scientific (Pty) Ltd. Cape Town, South Africa (SA). Foetal bovine serum (FBS) was purchased from Celtic Molecular Diagnostics, Cape Town, SA. Penicillin-streptomycin and phosphate-buffered saline tablets (Cat No. 524650) were from Merck (Pty) Ltd., SA. 1, 1- Diphenyl 2-picrylhydrazyl (DPPH) and L-ascorbic acid, Taxol and HPLC-grade methanol were purchased from Sigma Aldrich, SA. The Celltiter-GloTM cell viability assay kit and GSH-Glo™ glutathione assay reagents were purchased from Anatech, SA. OxiSelectTM TBARS assay kit (MDA Quantitation) (STA-33O) was purchased from Biocom, SA. The nonpyrogenic sterile filter system was obtained from Corning Incorporated (USA). The 96-well opaque plates and filtered pipette tips were purchased from Whitehead Scientific (Pty) Ltd., Cape Town, SA. Glomax Multi Detection System (model 9301-010) and the Zenyth 200rt UV-Vis spectrophotometer were used for detection. The chemicals used for the phytochemical screening were sulfuric acid, sodium phosphate, ammonium molybdate, ferric chloride, sodium hydroxide and hydrochloric acid and were all purchased from Sigma Aldrich, SA.

### Plant materials and the preparation of Emelia M, Mshikazi and Delosma H Extracts

The three traditional herbal medicines were provided by three traditional health practitioners (THPs) from KwaZulu-Natal, South Africa, in the year 2017. The EMB, Mshikazi and Delosma H decoctions were prepared in aqueous form according to the information gathered from the THPs and were provided as ready to use aqueous extracts. The names of the plants have been withheld to protect the intellectual property of the knowledge holders. However, the relevant fresh plant material was preserved with the assistance of a botanist for later verification when the intellectual issues were addressed.

The extracts were filter-sterilised using an Automatic Lid Clock (SP Scientific, USA) centrifuge at 3700 rpm for 10 minutes. After centrifugation, the supernatants were freeze-dried to powder separately using the SP scientific freeze dryer. All extracts were weighed, collected in vials and kept in a refrigerator (-20°C) for long term storage. A stock solution of each herbal extract was prepared by weighing 100 mg of the powdered material which then was dissolved in 10 ml of phosphate-buffered saline (PBS) to make a stock solution of 10 mg/mL. These stock solutions were further filter-sterilised using a corning filter bottle with a filter pore size of 0.22 µm. The half-maximum inhibitory concentration (IC50) for each extract was determined using peripheral blood mononuclear cells (PBMCs) isolated from normal human blood samples.

### Cell lines and cell culture

The Jurkat cell lines were kindly donated by Dr Bongiwe Ndlovu from the UKZN HIV Pathogenesis Programme and THP-1 monocyte cell lines were obtained from Mr Saiyur Ramsugit of the Discipline of Medical Microbiology, University of KwaZulu-Natal. PBMCs were provided by Dr Jacobus Hendricks, Human Physiology, School of Laboratory Medicine and Medical Sciences, UKZN. PBMCs, THP-1 monocyte and Jurkat lymphocytes were cultured in RPMI-1640 medium supplemented with 10% foetal bovine serum 1% L-glutamine and 1% penicillin-streptomycin at 37°C in humidified 5% CO_2_ atmosphere.

### Peripheral blood mononuclear cells (PBMC) viability assay

The effect of varying concentrations of the three extracts on the viability of PBMCs was separately evaluated using the ATP assay. PBMCs were seeded in 24-well plates at a density of 1.0 × 106 cells/well. Varying concentrations of Delosma H and EMB extracts (100, 250, 500 1000, 25000, 5000 and 7500 µg/mL) and Mshikazi (10, 25, 50, 75, and 100 µg/mL) were added and incubated at 37°C in humidified 5% CO2 atmosphere for a time range of 24. At the end of each incubation period, 100 µL from each treatment concentration was pipetted in triplicate into wells of a white opaque 96-well plate. The CellTiter-Glo reagent (Promega) (to determine the number of viable cells) was mixed immediately out of direct light before use and was added to the wells with treated cells, untreated cells and blank at 50 µL per well. The 96-well plates were then shaken on a plate shaker for 15 seconds at 250 rpm. The plate was incubated out of direct light for 10 minutes at room temperature. The 96-well plates containing the cells were put into the luminometer (GLOMAX multi-detection system 9301-010 model) and the relative light units (RLU) of the samples were measured to determine the amount of ATP in the viable cells. IC_50_ values for each of the three extracts of THMs was determined from this assay. The IC_50_ doses were then used to assess the effects of extracts on the viability of THP-1 monocytes, and Jurkat lymphocytes cell lines. To determine the IC_50_ value, concentrations of extracts were plotted against the percentage viability using the linear (y = mx+c) equation. On this graph, y = 50, x = IC_50_.

Linear equation: (y = mx+c)

(y-c)/m = x

Log of both sides

(y-c)/m = x

Where y = 50, c = constant, m = slope and x = IC_50_.

### 1, 1-Diphenyl-2-picrylhydrazyl (DPPH) radicals scavenging activity assay

The antioxidant activity of the three herbal medicines (Delosma H, Mshikazi and EMB) was evaluated based on the stable 1,1-diphenyl-2-picrylhydrazyl (DPPH) free radical according to a previously described method with minor modifications^18^. A volume of 200 µL of 200 µM DPPH solution in methanol (from 1000 µM stock DPPH solution in methanol) was mixed with 600 µL of each IC_50_ concentration of the extracts in methanol (Delosma H, 2268.7 µg/mL, Mshikazi, 134.3 µg/mL and EMB, 1954.0 µg/mL). L-Ascorbic acid (30µM) was used as a reference standard. DPPH (200 µM) in methanol was used as a control and methanol only was used as a blank. Samples and controls were then incubated in the dark at 37°C for 30 minutes. The absorbance of the reaction mixture was then measured in triplicate at 517 nm using a UV-Vis spectrophotometer. The inhibition percentage was calculated using the following formula:

% inhibition = (Ac-As)/Ac x 100

Where Ac is the absorbance of the control, As is the absorbance of the test sample.

### Glutathione assay

The GSH-Glo™ assay (Promega, SA) was used to measure GSH levels in THP-1 monocytes and Jurkat lymphocytes cell lines treated with the IC_50_ concentrations of EMB (1954.0 µg/mL), Mshikazi (134.3 µg/mL) and Delosma H (2268.7 µg/mL) and incubated at 24 and 48 h. Taxol at 20µM was used as a positive control and the untreated cells were also included as negative controls. At the end of each incubation period, 1 mL of the treated THP-1 and Jurkat cells were measured into Eppendorf tubes and centrifuge at 1500 rpm for 7 minutes. The cells were re-suspended in of PBS (250 µL) and 50 µL/well (1.0 × 107 cells/mL) and 10 µL/well of GSH standards (0–5 µM) were measured into separate triplicate wells of a 96-well opaque plate. Luciferin-NT-substrate (25 µL) and glutathione-S-transferase diluted to 1:50 in GSH-Glo™ (Reaction Buffer) was added in each well and incubated for 30 minutes at room temperature in the dark. Thereafter, 50 µL of luciferin detection reagent was added and the plate was incubated for 15 minutes at room temperature in the dark. The luminescence was measured using a luminometer (Glo-Max multi-detection system model 9301-010, Promega, USA).

### Phosphomolybdate assay

To determine the total antioxidant capacity (TAC) of EMB, Mshikazi and Delosma H extracts, the procedure described by Ahmed, Khan and Saeed^10^ and Khatoon et al^19^ was used with minor modifications. The IC_50_ concentrations for each extract were prepared from the stock. Ascorbic acid (30µM) was used as a standard antioxidant reference. In a test tube, 300 µL of each extract or ascorbic acid was mixed with 3 mL phosphomolybdate reagent (0.6 M sulfuric acid, 28 mM sodium phosphate and 4 mM ammonium molybdate). The test tube was then placed in a water bath at 95°C for 90 minutes. The mixture was then allowed to reach room temperature and the absorbance was recorded at 695 nm against the methanol blank using a spectrophotometer. The experiment was carried out in triplicate and the antioxidant capacity was calculated. The result was expressed as microgram of ascorbic acid equivalent (AAE) per millilitre^10^,^19^.

### Lipid peroxidation (LPO) activity

The lipid peroxidation activity of the extracts were evaluated using the thiobarbituric acid reactive substance (TBARS) assay. This TBARS method is based on the spectrophotometer measurement of the pink colour produced through the reaction of thiobarbituric acid (TBA) with malondialdehyde (MDA) and other secondary lipid peroxidation products^20^. TBARS was determined following the protocol in the TBARS assay kit (OXiSelect TM TBARS assay kit, MDA quantitation). Standard MDA solutions were prepared by serial dilution in the concentration ranging from 125 to 0 µM by diluting the MDA standard in deionized water. Treated THP-1 and Jurkat cells supernatants (100 µL) were pipetted into separate Eppendorf tubes followed by the addition of 1 µL of 100 x 5% butylated hydroxytoluene and centrifuged at 15000 rpm for 10 minutes. SDS lysis solution (100 µL) was added to the samples and the MDA standard and reactions were mixed and incubated for 5 minutes at room temperature. TBA reagent (250 µL) was added to samples and standards and incubated at 950C for 45 minutes. The resulting reaction was then cooled for 5 minutes on ice. The samples and standard were then centrifuged at 3000 rpm for 15 minutes. Samples and standard (150 µL) were pipetted into duplicate wells of a clear 96-well microplate and read at 532 nm on a spectrophotometer (Zenyth 200rt UV-Vis Spectrophotometer).

### Phytochemical screening

The phytochemical screening of the extract of EMB, Mshikazi and Delosma H were conducted to detect the presence of phenolics, flavonoids, alkaloids, saponins, tannins, and terpenoids which are known to have several biological activities such as antioxidant, antimicrobial and anticancer properties^21^. The results are stated as (+) for the presence and (-) for the absence of phytochemical components.

### Test for terpenoids

The Salkowski test was used to determine terpenoids. EMB, Mshikazi and Delosma H extracts (about 50 mg each) were mixed with 3mL of chloroform and concentrated sulfuric acid (3mL) was added dropwise to each extract. The formation of a reddish-brown layer indicates the presence of terpenoids^21^,^22^.

### Test for phenolics

The ferric chloride test was used to determine the phenolics content of the extracts. Distilled water (5 mL) was added to each extract (50 mg) of EMB, Mshikazi and Delosma H in test tubes. Few drops of 1% ferric chloride (FeCl3) solution was added to the test tubes and allowed to mix. A change in colour to bluish-black indicates the presence of phenols^21^,^23^.

### Test for flavonoids

The alkaline reagent test was used to determine the flavonoid content of the extract. A volume of 1mL of each of the extract of EMB, Mshikazi and Delosma H was mixed with few drops of 10% sodium hydroxide (NaOH) solution in separate test tubes. The development of an intense yellow colour, which becomes colourless when diluting with hydrochloric acid indicates the presence of flavonoids^21^,^23^.

### Test for saponins

The Froth test was used to determine the saponin content of the extracts. Each extract (0.5 mg) was mixed thoroughly with 2 mL of distilled water in test tubes. A consistent formation of froth for 10 minutes indicates the presence of saponins^21^,^23^.

### Test for tannins

The test for tannins was determined by measuring 0.5 mg of the extracts which were dissolved in distilled water in a test tube. The mixed solutions were filtered with Whatman Number 1 filter paper. The filtrate (1 mL) was treated with 1% ferric chloride (FeCl3) solution dropwise. Blackish-blue or blackish green colour indicates the presence of tannins^21^.

### Test for Alkaloids

The test for alkaloid was determined by mixing the extracts with 6 mL of 1% hydrochloric acid that was heated gently in a water bath for 5 minutes and filtered. Mayer's reagent (1 mL) (potassium mercuric iodide solution) was added to the filtrate. A cream coloured precipitate indicates the presence of alkaloids^22^.

### Statistical analysis

Data analyses were done on Microsoft Excel (Microsoft Corporation, City, USA) to obtain descriptive statistics. The different levels of significances within the separate treated groups of the extracts for GSH were analysed using one-way analysis of variance (ANOVA) and the differences between the treated cells and the control cells were analysed using GraphPad Prism software (version 5) with the Tukey-Kramer multiple comparison test. Differences with P ≤ 0.05 were considered statistically significant.

## Results

### 1,1-Diphenyl-2-picrylhydrazyl (DPPH) radical scavenging activity

The free radical scavenging activity of the extract of the three traditional herbal medicines (EMB, Mshikazi, Delosma H) and a reference standard (L-ascorbic acid) was determined by their ability to reduce DPPH. L-Ascorbic acid (30µM) was used as a positive control. The results showed EMB to have the highest scavenging activity (92.8%) which was comparable to L-ascorbic acid (96.7%). The scavenging activity of Delosma H was 85.8% and of Mshikazi 74.5% relative to L-ascorbic acid. The scavenging activity was found to be in decreasing order of EMB < Delosma H < Mshikazi (Table 1).

**Table 1:** Inhibition of DPPH by *Emelia M, Mshikazi* and *Delosma H* and the reference ascorbic acid

Traditional herbal medicine & reference	% Inhibition of DPPH
L-Ascorbic acid (30 µM)	96.7
*Emelia M* (1954.0 µg/mL)	92.8
*Delosma H* (2268.7 µg/mL)	85.8
*Mshikazi* (134.3 µg/mL)	74.5

### Glutathione (GSH) assay

**Figure 1:**
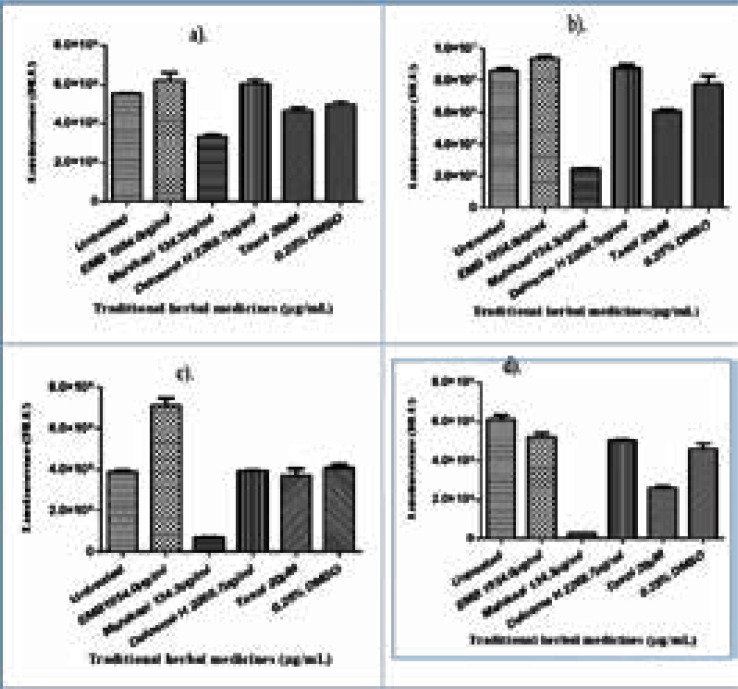
a) GSH levels of THP-1 cells at 24 h, b) GSH levels of Jurkat cells at 24 h, c) GSH levels of THP-1 cells at 48 h, d). GSH levels of Jurkat cells at 48 h of incubation for *Emelia M* (EMB), *Mshikazi, Delosma H* and Taxol (positive control).

Figure 1a) GSH levels of THP-1 cells at 24 h, b) GSH levels of Jurkat cells at 24 h, c) GSH levels of THP-1 cells at 48 h, d). GSH levels of Jurkat cells at 48 h of incubation for Emelia M (EMB), Mshikazi, Delosma H and Taxol (positive control).

### Delosma H

The levels of GSH in the THP-1 monocytes and Jurkat lymphocytes treated with Delosma H, Mshikazi and EMB were evaluated after 24 and 48 h of incubation. At 24 h incubation, GSH levels of Delosma H extract treated THP-1 cells increased significantly when compared to the controls (untreated and Taxol), P = 0.0008 (Figure 1a). At 48 h, Delosma H extract did not show a significant change in GSH levels for THP-1 cells when compared to the untreated control and Taxol (P = 0.6428, Figure 1c). With Jurkatells, at 24 h, Delosma H extract significantly increased GSH levels when compared to the controls (untreated and Taxol), P = 0.0007 (Figure 1b). There was a significant difference between Taxol (positive control) and the untreated control. At 48 h, Delosma H extract showed a significant decrease in GSH levels for Jurkat cells compared to the untreated control and Taxol (P = 0.0001, Figure 1d). There was a significant difference in the GSH level of Taxol (positive control) when compared to the untreated control.

### Mshikazi

At 24 h, the Mshikazi extract induced a decrease in the GSH levels of THP-1 compared to the controls (untreated and Taxol), P < 0.0001(Figure 1a). There was a significant difference in the GSH level of the Mshikazi extract when compared with the untreated control and Taxol (positive control). A similar trend was shown after 48 h of incubation, where the Mshikazi extract showed a significant decrease in GSH levels for THP-1 cells when compared to the untreated control and Taxol, P > 0.0001(Figure 1c). After 24 h of treatment with Mshikazi extract, Jurkat cells decreased GSH levels compared to the controls (untreated and Taxol), P < 0.0001(Figure 1b). A similar trend was shown after 48 h of incubation, where the Mshikazi extract showed a significant decrease in GSH levels for Jurkat cells when compared to the untreated control and Taxol, P > 0.0001(Figure 1d).

### Emelia M (EMB)

After 24 h incubation, GSH levels for the EMB extract treated with THP-1 cells increased significantly when compared to the controls (untreated and Taxol), P = 0.0082 (Figure 1a). A similar trend occurred at 48 h, where the EMB extract increased GSH levels in THP-1 cells compared to the controls (untreated and Taxol), P < 0.0001 (Figure 1c). At 24 h of treatment, EMB extracts increased the GSH levels of Jurkat cells when compared to the controls (untreated and Taxol), P = 0.0002 (Figure 1b). At 48 h, the EMB extract increased GSH levels of Jurkat cells when compared to the controls (untreated and Taxol), P < 0.0001 (Figure 1d). There was a significant difference between the GSH levels of the EMB extract-treated cells when compared to Taxol (positive control), while there was no significant difference with the EMB extract and untreated cells (Figure 6b). The glutathione levels were found to be in increasing order of EMB > Delosma H > Taxol > Mshikazi (Figure 1).

### Phosphomolybdate assay

**Figure 2:**
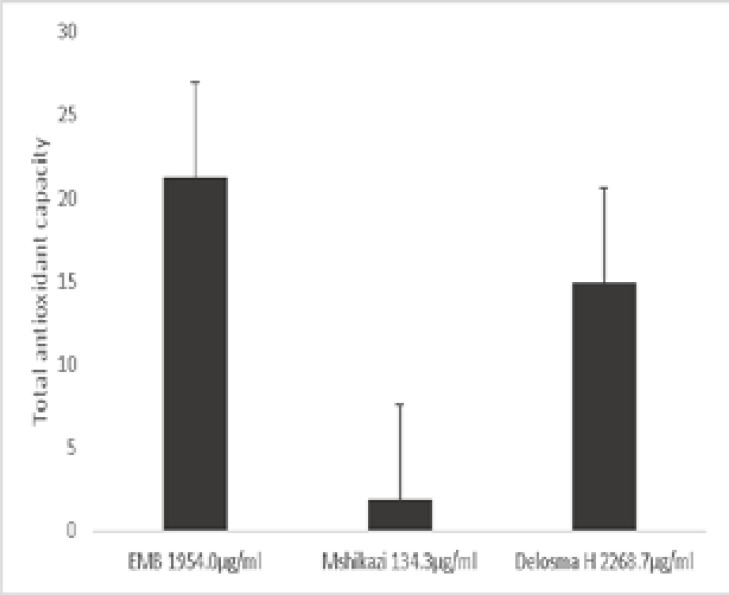
Phosphomolybdate reduction assay of *Emelia M* (EMB), *Mshikazi* and *Delosma H* extracts showing the antioxidant activity after heating for 90 minutes, values (in µg of ascorbic acid equivalent (AAE) per mL) are mean plus SEM (n=3).

This assay is based on the reduction of phosphomolybdate ion, where molybdenum(V1) is reduced to molybdenum(V) in the presence of an antioxidant resulting in the formation of a green phosphomolybdate(V) complex which can be determined at 695 nm^10^,^19^. This assay involves an electron transfer mechanism. The results of the total antioxidant activity of the extracts and a known antioxidant, ascorbic acid is showed in Figure 2. EMB had higher antioxidant activity than the other two extracts (Delosma H and Mshikazi). The order of the antioxidant activity was EMB > Delosma H > Mshikazi (Figure 2).

### Lipid peroxidation

**Figure 3:**
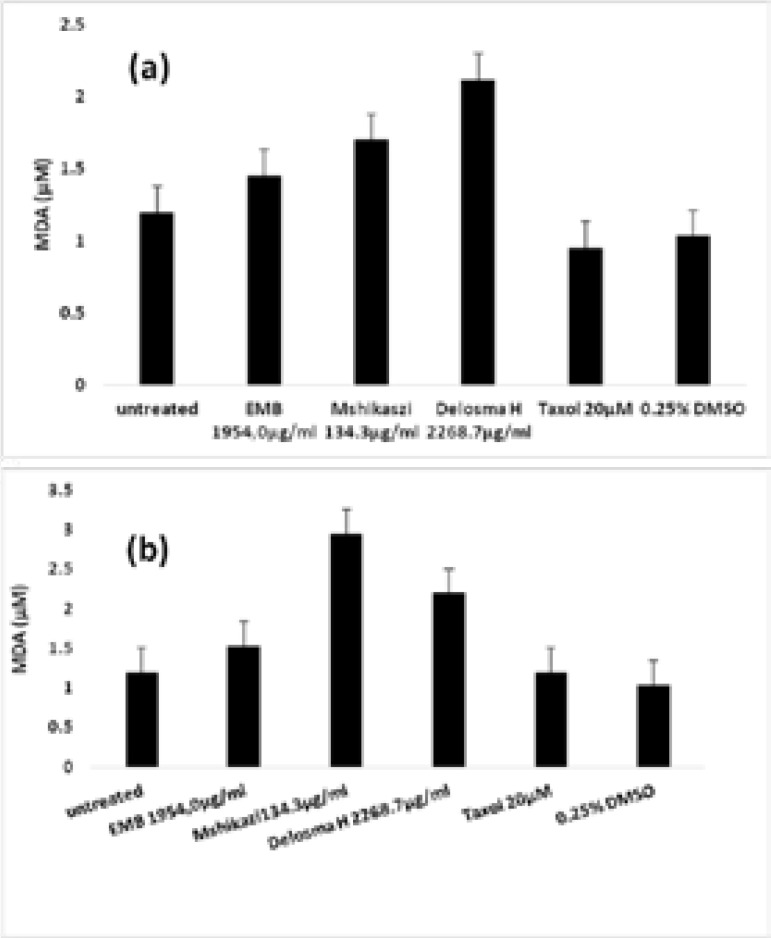
Lipid peroxidation activity of *Emelia M* (EMB), *Mshikazi* and *Delosma H* extracts on THP-1 (a) and Jurkat (b) cells after 24 h. Values are mean plus SEM (n=3).

The lipid peroxidation activity of EMB, Mshikazi and Delosma H extracts were examined on THP-1 and Jurkat cells in vitro using the thiobarbituric acid reactive substance (TBARS) assay. The results showed that Delosma H, Mshikazi and EMB extracts induced lipid peroxidation of both THP-1 and Jurkat cells compared to the untreated cells and Taxol after 24 h of treatment. The order of the induced lipid peroxidation in THP-1 cells by the traditional herbal medicines was Delosma H > Mshikazi > EMB shown in Figure 3a while the order in Jurkat cells was Mshikazi > Delosma H > EMB has shown in Figure 3b.

### Phytochemical screening

Phytochemical components of the extracts of EMB, Mshikazi and Delosma H were determined using various standard screening tests. The results of the phytochemical components present in the three extracts are shown in Table 2. The results show phenols, flavonoids, saponins and terpenoids to be present in all the three extracts and tannins to be absent in Mshikazi. All extracts tested negative for alkaloids.

**Table 2:** The phytochemical screening of *Emelia M* (EMB), *Mshikazi* and *Delosma H* extracts

Classes	Positive Results	EMB	*Mshikazi*	*Delosma H*
**Phenols**	bluish black	+	+	+
**Flavonoids**	Intense yellow	+	+	+
**Saponins**	Froth formation	+	+	+
**Tannins**	Blue-black or green-black	+	-	+
**Terpenoids**	Reddish-brown	+	+	+
**Alkaloids**	Red precipitate	-	-	-

(+) indicates the presence of the components and (-) indicates the absence of the components.

## Discussion

Free radicals are chemicals that occur separately and have one or more unpaired valence electrons. These unpaired electrons make free radicals highly reactive^4^. The free radicals are formed in the body through internal or external factors which are liable to many diseases. The proliferative effects of free radicals can lead to adverse effects that cause damage to cells^4^,^24^. Antioxidants have health benefits such as protecting the body system against free radicals or reactive oxygen species to fight against diseases. Research has been ongoing to reveal the potential of medicinal plant species that possess antioxidant activities since synthetic antioxidants have been shown to cause harm to the body system^24^. Plants are a source of natural antioxidants which are known to be healthy and safe.

In this study, the antioxidant activity and phytochemical constituents of EMB, Mshikazi and Delosma H herbal extracts were evaluated. Scavenging activities of the herbal extracts against DPPH radicals show that the scavenging activities are due to the electron transfer/ability to donate hydrogen atom^25^. Ascorbic acid (the standard), EMB, Mshikazi and Delosma H herbal extracts possess varying scavenging activities. The extracts of EMB and Delosma H, which showed free radical scavenging activity comparable to ascorbic acid, possibly contains compounds that can donate hydrogen to the free radical to stabilise it. These herbal extracts which have antioxidant capacity comparable to ascorbic acids such as EMB and Delosma H show that there is the presence of free radical inhibitor perhaps behaving like primary antioxidants^25^. Previous studies highlight the antioxidant and free radical scavenging activities of decoctions and ethanolic extracts of the leaves of Philippine medicinal plants (F. nota, M. sagu, M. philippica, I. fagifer, and C. mercadoi) using two in vitro antioxidant assays^26^. The methanolic extracts of C. mercadoi were shown to have the highest total phenolic content which was likely to have contributed to its strong antioxidant activity^17^. More also, DPPH radical scavenging activity was used to evaluate twelve medicinal herbals to determine their antioxidant activity using Ascorbic acid as a standard., the results show that the ascorbic and these medicinal herbs Atropa acuminata, Crocus sativus Carthamus tinctorius and Picrorrhiza kurroa have the highest antioxidant activity compared to others herbs^27^. These findings were comparable to ours where EMB and Delosma H herbal extracts have the highest antioxidant activity which was comparable with ascorbic acid.

The total antioxidant capacity of the three herbal extracts was further evaluated using the phosphomolybdate reduction assay which involves an electron transfer mechanism and a reduction of molybdenum(VI) to molybdenum(V)^10^. This assay showed that the herbal extracts contain antioxidants that reduce Mo(VI) to Mo(V), the complexes of which are green^28^. The high activity exhibited by these herbal extracts especially EMB, which was higher than the other two herbal extracts and ascorbic acid, similar to the DPPH assay, show that EMB extract is rich in antioxidants. Our results for these active herbal extracts correspond with the study on methanol extracts of Azadirachta indica and M. charantia at 1000 µg/mL which were reported to show strong radical scavenging activity (96.50% and 95.25%, respectively)^29^. This indicates that these extracts possess high amounts of antioxidants which can fight against diseases, boost the immune system and reduce the risk of infection with cancer patients. A report by Gaman et al.^30^ showed that a healthy lifestyle which is rich in natural antioxidants can reduce the risk of infectious diseases and may improve the quality of life for patients living with leukaemia.

To evaluate the effects of the three herbal extracts on intracellular antioxidants, changes in glutathione (GSH) levels were quantified using a luminometry-based assay. GSH is an antioxidant which acts as a free radical scavenger and a purification agent in cells. With cancer, it performs a dual function in its progression such as elimination and detoxification of carcinogens^31^. GSH also plays a useful role in many cellular processes which include cell growth, cell differentiation and programmed cell death^32^. A decrease/reduction in GSH will lead to an increase in oxidative stress, which is associated with the progression of cancer but an elevated GSH level will increase the capacity of the antioxidant thereby causing resistance in oxidative stress^32^. EMB and Delosma H significantly increased the intracellular levels of GSH in THP-1 cells and Jurkat cell at 24 h but not at 48 h. This indicates that thherbal extracts have high antioxidant capacity which can cause resistance to oxidative stress, boost the immune system and resist further growth of the ROS. Previous studies have shown a positive correlation between cell growth and GSH because of this antioxidant fights against ROS, mutagens and drugs^33^. A study by Naidoo et al^17^ showed that Centella asiatica extract increased the GSH concentration of THP-1 cells at 72 h while in this study, the EMB and Delosma H extracts were effective in shorter treatment times. The Mshikazi extract and the positive control (Taxol), on the other hand, caused a significant reduction in the intracellular GSH levels of THP-1 and Jurkat, indicating low antioxidant capacity. The decrease in GSH concentration makes the cancer tumour cells more liable to ROS thereby inducing cell death. In our anticancer study on Mshikazi, we have shown that this extract induced high anti-proliferative effects on both THP-1 and Jurkat cells (data not shown). A previous study^34^ using Tulbaghia violacea Harv leaf and stalk extracts showed a significant increase in GSH production of Jurkat cells. The cell growth of Jurkat T-cells was lower when treated with the methanol extract of T. welwitschii roots and GSH at 72 h^33^. Futhermore, the increase in GSH levels by these herbal extracts, EMB and Delosma H extracts corresponds with observed high free radical scavenging activity in the DPPH assay. It has been reported by Moyo and Mukanganyama^33^ and Syng-ai et al^35^ that cancer cells have more intracellular GSH than normal cells and the reduction in GSH concentration make the cancer cells more prone to ROS which causes cell death. There is, therefore, a possibility that Mshikazi induced cell death (apoptosis) on the THP-1 and Jurkat cells through glutathione (GSH) depletion.

An increase in free radicals / reactive oxygen species can exact direct damage to lipids^36^. Lipid peroxidation is said to be the main molecular mechanism that is involved in the oxidative damage to cell structures and in the toxicity process that leads to cell death^37^. the treatment option can either generate ROS thereby causing oxidative stress (prooxidant) or reduced ROS thereby reducing the causes of oxidative stress (antioxidant). A slight increase in lipid peroxidation of THP-1 and Jurkat cells treated with EMB, and significant increase with Mshikazi, and Delosma H extracts, show that these herbal extracts induced ROS, therefore act as a prooxidant and induced cell damage to the leukaemia cell lines which lead to cell death. In a review, it was stated that when there are low lipid peroxidation rates (nontoxic conditions), the cells stimulate their maintenance and survival through antioxidant defence systems whereas at high lipid peroxidation rates (toxic conditions) the extent of oxidative damage overpowers repair capacity, and the cells induce apoptosis or necrosis programmed cell death^36^. The latter process is what occurs to the leukaemia cells after treating with these herbal extracts. A study by Gaschler and Stockwell^38^ reported that lipid peroxidation played a role in regulated cell death and the degradation of 4-hydroxynonenal during this process has been shown to instigate apoptosis^39^.

Therefore, EMB, Mshikazi, and Delosma H extracts play a dual role effect, as an antioxidant, as seen by some assays report and as well as a prooxidant in the case of lipid peroxidation. Ascorbic acid was said to have both antioxidant and prooxidant effects depending upon the dose^9^.

Phytochemical screening was used to determine the secondary metabolites present in the three extracts (EMB, Mshikazi and Delosma H). These constituents included phenols, saponins, terpenoids, flavonoids and tannins; alkaloids were absent in all extracts. These antioxidant compounds have health benefits on both cancer and other chronic diseases.

Cancer and other chronic diseases have been a global health concern and cause millions of deaths globally^40^,^41^. It has been reported that fruits, vegetables and grains has a protective effect against the development of cancer and other chronic diseases; these protective effects have been ascribed to the antioxidant phytochemicals which are presents in them^40^,^41^. Antioxidant phytochemicals are referred to as bioactive non-nutrient plant compounds that have been shown to reduce the risk of cancer caused by oxidative stress^40^–^42^. These antioxidant phytochemicals which are phenols, saponins, terpenoids, flavonoids, tannins and alkaloids are secondary metabolites and have been shown to have importance in the treatment of cancer and boosting immune system^41^,^43^,^44^.

Phenolic constituents are the largest group of plant secondary metabolites and have biological properties such as antioxidant, anti-apoptosis, anticancer and anti-inflammatory, and may decrease the risk of heart disease^21^,^23^. Several studies have reported cytotoxic and apoptosis properties of medicinal plants which are rich in phenols^21^,^45^.

The extracts of EMB, Mshikazi and Delosma H were shown to also have flavonoids which are derivatives of phenolic constituents that have antioxidant, antitumor and anti-inflammatory properties^21^. Saponins are known to possess anticancer, antimicrobial and anti-inflammatory properties^21^. Tannins are said to have antioxidant, antibacterial and antiviral activities^21^. The absence of tannins in Mshikazi explains the low antioxidant activity of this extract as shown through the DPPH and phosphomolybdate reduction assays. EMB, Mshikazi and Delosma H extracts were also shown to contain terpenoids. Natural triterpenes, sesquiterpenes and diterpenes are terpenoids that possess antioxidant, anticancer, anti-inflammatory, and enzyme inhibitory activities^46^. The presence of the compounds found in these three herbal extracts may have antioxidant/prooxidant properties. With the antioxidant properties, these compounds found may be said to acts as a supplement to aid immunity and boost the production of detoxifying enzymes in the body and increasing the intracellular protein thiol molecule in the leukaemia cells, thereby preventing cell damage caused by the ROS. Apart from the antioxidant properties exerted by the phytochemicals from herbal extracts, they also play a role as prooxidants, which indicate that these compounds found in the herbal extracts can also possess an anticancer properties, .thereby inhibiting the growth of the leukaemia cells.

Studies have shown different medicinal plants that possess phytochemical compounds such as flavonoids, saponins, tannins and terpenoids to have antioxidant and anticancer activities which correspond with the findings in this study. These plants include Andrographis paniculata, Aegle marmelos, Glycyrrhiza glabra^45^, Sarcocarp of C. multiflorus^6^, Goniothalamus velutinus^22^, Myrianthus arboreus^47^, Adiantum caudatum^10^, Zanthoxylum capense^48^, Laurencia majuscule, Padina pavonica^49^ and Alphonsea sclerocarpa^8^. A study on the bioactive principles from Zanthoxylum capense (small knobwood) reported on the isolation of alkaloids, coumarates, lignans, flavonoids, triterpenes and pigment molecules. The evaluated compounds showed synergistic effects for free radical scavenging activity and antagonistic effects for cytotoxicity as the isolated compounds significantly reduced the viability of MCF-7 tumour cells. In another study, the polyphenol content and antioxidant capacity of Myrianthus arboreus root extracts were evaluated which showed the ethanol extract to be effective antioxidants, more than BHT and Oligopin®. The phenolic, hydroxycinnamic acid and proanthocyanidin content in M. arboreus was comparable to Oligopin® which may have attributed to the antioxidant activity^47^. A study was conducted to evaluate the comparative phytochemical analysis and antioxidant activities of Tamalakyadi decoction with its modified dosage forms^50^. The phytochemical screening showed saponins, alkaloids, flavonoids, terpenoids, and steroids to be more prominent in the decoction that was freeze-dried compared to the other two preparations. The antioxidant activity confirmed the freeze-dried preparation to have higher radical scavenging ability compared to spray-dried and ganasara preparations. Freeze-dried decoctions were found to be the most suitable ready-touse preparation due to similar chemical properties to Tamalakyadi decoction (starting material). The results show the extracts of EMB, Mshikazi and Delosma H to be potential sources of natural antioxidants and has the potential to boost the immune system and combat free radical degenerative diseases.

## Conclusion

Based on the results obtained in this study, it is evident that EMB and Delosma H extracts of traditional herbal medicines have the most effective free radical scavenging properties compared to the Mshikazi extract. The herbal extracts also increased the level of intracellular protein thiol molecule in the leukaemia cells through glutathione assay. All three extracts of traditional herbal medicines increased lipid peroxidation and possess phytochemical components which have potent antioxidant and prooxidant properties. (increase in lipid peroxidation) activities. These properties they possess make the extracts to act as an immune booster as well as causing cell death on the leukaemia cells. More research should be conducted on these herbal extracts such as In vivo and observational studies on the patients taking the herbal medicines.

## References

[1] Saha SK, Lee S Bin, Won J, Choi HY, Kim K, Yang GM (2017). Correlation between oxidative stress, nutrition, and cancer initiation. Int J Mol Sci.

[2] Zhang J, Lei W, Chen X, Wang S, Qian W (2018). Oxidative stress response induced by chemotherapy in leukemia treatment (Review). Mol Clin Oncol [Internet].

[3] Saed GM, Diamond MP, Fletcher NM (2017). Updates of the role of oxidative stress in the pathogenesis of ovarian cancer. Gynecol Oncol [Internet].

[4] Partap S, Tewari U, Sharma K, Jha KK (2014). In Vitro Antioxidant Activity of Whole Plant of Leptadenia Pyrotechnica. J Drug Delievery Ther.

[5] Bajpai VK, Agrawal P, Park YH (2014). Phytochemicals, Antioxidant and Anti-lipid peroxidation Activities of Ethanolic extract of a Medicinal plant, Andrographis paniculata. J Food Biochem.

[6] Liu X, Jia J, Jing X, Li G (2018). Antioxidant Activities of Extracts from Sarcocarp of Cotoneaster multiflorus. J Chem.

[7] Gupta RK, Patel AK, Shah N, Choudhary AK, Jha UK, Yadav UC (2014). Oxidative Stress and Antioxidants in Disease and Cancer: A Review. Asian Pacific J Cancer Prev [Internet].

[8] Dsd SJ, Amgoth C, S SN, Ch P, Madhavi J, A KS (2018). Antioxidant and anticancer activities of an Aporphine alkaloid isolated from Alphonsea sclerocarpa. J Phytopharm.

[9] Rahal A, Kumar A, Singh V, Yadav B, Tiwari R, Chakraborty S (2014). Oxidative Stress, Prooxidants, and Antioxidants: The Interplay. Biomed Res Int.

[10] Ahmed D, Khan M, Saeed R (2015). Comparative Analysis of Phenolics, Flavonoids, and Antioxidant and Antibacterial Potential of Methanolic, Hexanic and Aqueous Extracts from Adiantum caudatum Leaves. Antioxidants [Internet].

[11] Kumar AP, Graham H, Robson C, Garapati K, Ghosh R (2011). An Overview of Anticancer Herbal Medicines.

[12] Mahomoodally MF (2013). Traditional Medicines in Africa: An Appraisal of Ten Potent African Medicinal Plants. Evidence-based Complement Altern Med.

[13] Yatoo MI, Gopalakrishnan A, Saxena A, Parray OR, Tufani NA, Chakraborty S (2018). Anti-Inflammatory Drugs and Herbs with Special Emphasis on Herbal Medicines for Countering Inflammatory Diseases and Disorders - A Review. Bentham Sci.

[14] Bhatt ID, Rawat S, Rawal R (2013). Antioxidants in Medicinal Plants.

[15] Yagi SM, Yagi AI (2018). Traditional medicinal plants used for the treatment of diabetes in the Sudan: A review. African J Pharm Pharmacol.

[16] Kadan S, Saad B, Sasson Y, Zaid H (2013). In Vitro Evaluations of Cytotoxicity of Eight Antidiabetic Medicinal Plants and Their Effect on GLUT4 Translocation. Evidence-based Complement Altern Med.

[17] Naidoo DB, Chuturgoon AA, Phulukdaree A, Guruprasad KP, Satyamoorthy K, Sewram V (2017). Centella asiatica modulates cancer cachexia associated inflammatory cytokines and cell death in leukaemic THP-1 cells and peripheral blood mononuclear cells (PBMC's). BMC Complement Altern Med [Internet].

[18] Sahu RK, Kar M, Routray R (2013). DPPH Free Radical Scavenging Activity of Some Leafy Vegetables used by Tribals of Odisha, India. J Med Plant Stud.

[19] Khatoon M, Islam E, Islam R, Rahman AA, Alam AHMK, Khondkar P (2013). Estimation of total phenol and in vitro antioxidant activity of Albizia procera leaves. BMC Res Notes.

[20] Boubaker J, Bhouri W, Sghaier M, Bouhlel I, Skandrani I, Ghedira K (2011). Leaf extracts from Nitraria retusa promote cell population growth of human cancer cells by inducing apoptosis. Cancer Cell Int [Internet].

[21] Nordin ML, Abdul Kadir A, Zakaria ZA, Othman F, Abdullah R, Abdullah MNH (2017). Cytotoxicity and Apoptosis Induction of Ardisia crispa and Its Solvent Partitions against Mus musculus Mammary Carcinoma Cell Line (4T1). Evidence-based Complement Altern Med.

[22] Iqbal E, Salim KA, Lim LBL (2015). Phytochemical screening, total phenolics and antioxidant activities of bark and leaf extracts of Goniothalamus velutinus (Airy Shaw) from Brunei Darussalam. J King Saud Univ - Sci.

[23] Khanam Z, Wen CS, Bhat IUH (2015). Phytochemical screening and antimicrobial activity of root and stem extracts of wild Eurycoma longifolia Jack (Tongkat Ali). J King Saud Univ - Sci.

[24] Ansari AQ, Ahmed SA, Waheed MA, A SJ, Abrar S (2013). Extraction and determination of antioxidant activity of Withania somnifera Dunal. Eur J Exp Biol.

[25] Shabbir M, Khan MR, Saeed N (2013). Assessment of phytochemicals, antioxidant, anti-lipid peroxidation and anti-hemolytic activity of extract and various fractions of Maytenus royleanus leaves. BMC Complement Altern Med.

[26] Latayada FS, Uy MM (2016). Screening of the Antioxidant Properties of the Leaf Extracts of Philippine Medicinal Plants Ficus nota (Blanco) Merr ., Metroxylon sagu Rottb., Mussaenda philippica A. Rich., Inocarpus fagifer, and Cinnamomum mercadoi Vidal. Bull Environ Pharmacol Life Sci.

[27] Rashid S, Ahmad M, Zafar M, Anwar A, Sultana S, Tabassum S (2016). Ethnopharmacological evaluation and antioxidant activity of some important herbs used in traditional medicines. J Tradit Chinese Med [Internet].

[28] Olugbami J, Gbadegesin M, Odunola O (2015). In vitro evaluation of the antioxidant potential, phenolic and flavonoid contents of the stem bark ethanol extract of Anogeissus leiocarpus. Afr J Med Med Sci.

[29] Patel R, Patel Y, Kunjadia P (2015). Original Research Article DPPH free radical scavenging activity of phenolics and flavonoids in some medicinal plants of India. International J Curr Microbiol Appl Sciences.

[30] Gaman AM, Buga AM, Gaman MA, Popa-Wagner A (2014). The role of oxidative stress and the effects of antioxidants on the incidence of infectious complications of chronic lymphocytic leukemia. Oxid Med Cell Longev.

[31] Bansal A, Simon MC (2018). Glutathione metabolism in cancer progression and treatment resistance. J Cell Biol.

[32] Traverso N, Ricciarelli R, Nitti M, Marengo B, Furfaro AL, Pronzato MA (2013). Role of Glutathione in Cancer Progression and Chemoresistance. Oxid Med Cell Longev.

[33] Moyo B, Mukanganyama S (2015). Antiproliferative Activity of T. welwitschii Extract on Jurkat T Cells In Vitro. Biomed Res Int.

[34] Mackenzie J, Moodley K, Mackraj I, Chuturgoon A, Phulukdaree A, Serumula M (2017). Apoptosis-inducing effects of Tulbaghia violacea Harv methanolic extracts on human Jurkat leukemia T cells. Indian J Tradit Knowl.

[35] Syng-ai C, Leela Kumari A, Khar A (2004). Effect of curcumin on normal and tumor cells: Role of glutathione and bcl-2 Effect. Mol Cancer Ther.

[36] Ayala A, Muñoz MF, Argüelles S (2014). Lipid Peroxidation: Production, Metabolism, and Signaling Mechanisms of Malondialdehyde and 4-Hydroxy-2-Nonenal. Oxid Med Cell Longev.

[37] Repetto M, Semprine J, Boveris A (2012). Lipid Peroxidation: Chemical Mechanism, Biological Implications and Analytical Determination. J Free Radic Biol Med [Internet].

[38] Gaschler MM, Stockwell BR (2017). Lipid peroxidation in cell death. Biochem Biophys Res Commun [Internet].

[39] Dalleau S, Baradat M, Guéraud F, Huc L (2013). Cell death and diseases related to oxidative stress:4-hydroxynonenal (HNE) in the balance. Cell Death Differ.

[40] Zhang YJ, Gan RY, Li S, Zhou Y, Li AN, Xu DP (2015). Antioxidant phytochemicals for the prevention and treatment of chronic diseases. Molecules.

[41] Meybodi NM, Mortazavian AM, Monfared AB, Meybodi FA (2017). Phytochemicals in Cancer Prevention: A Review of the Evidence. Iran J Cancer Prev.

[42] Liu RH (2004). Nutrition, and Cancer Potential Synergy of Phytochemicals in Cancer Prevention: Mechanism of Action. Am Soc Nutr Sci.

[43] Kamal A (2014). Phytochemical screening of syzygium cumini seeds. Indian J plant Sci.

[44] T JS, Poompavai S, Smf MB, V GS, Hemalatha S, Sieni E (2019). Cancer-Fighting Phytochemicals: Another Look. J Nanomedicine ad Biother Discov.

[45] Modi HA, Jain NK (2016). Assessment of anticancer properties of few medicinal plants. J Pharmacogn phytocheimstry.

[46] Rufino-Palomares EE, Perez-Jimenez A J, Reyes-Zurita F, Garcia-Salguero L, Mokhtari K, Herrera-Merchan A (2015). Anti-cancer and Anti-angiogenic Properties of Various Natural Pentacyclic Tri-terpenoids and Some of their Chemical Derivatives. Curr Org Chem [Internet].

[47] Kasangana P, Haddad P, Stevanovic T (2015). Study of Polyphenol Content and Antioxidant Capacity of Myrianthus Arboreus (Cecropiaceae) *Root Bark Extracts*. Antioxidants [Internet].

[48] Bodede O, Moodley R, Shaik S, Singh M (2016). Phytochemical Analysis with Antioxidant and Cytotoxicity Studies of the Bioactive Principles from Zanthoxylum capense (Small Knobwood). Anticancer Agents Med Chem.

[49] Al-Enazi NM, Awaad AS, Zain ME, Alqasoumi SI (2017). Antimicrobial, antioxidant and anticancer activities of Laurencia catarinensis, Laurencia majuscula and Padina pavonica extracts. Saudi Pharm J [Internet].

[50] Dahanayake JM, Perera PK, Galappatty P, Dona H, Melshandi S, Dona L (2019). Comparative Phytochemical Analysis and Antioxidant Activities of Tamalakyadi Decoction with Its Modified Dosage Forms. Evidence-based Complement Altern Med.

